# TIM-3 Suppresses Anti-CD3/CD28-Induced TCR Activation and IL-2 Expression through the NFAT Signaling Pathway

**DOI:** 10.1371/journal.pone.0140694

**Published:** 2015-10-22

**Authors:** Brian Tomkowicz, Eileen Walsh, Adam Cotty, Raluca Verona, Nina Sabins, Fred Kaplan, Sandy Santulli-Marotto, Chen-Ni Chin, Jill Mooney, Russell B. Lingham, Michael Naso, Timothy McCabe

**Affiliations:** Janssen BioTherapeutics, 1400 McKean Road, Spring House, PA 19477, United States of America; Jackson Laboratory, UNITED STATES

## Abstract

TIM-3 (T cell immunoglobulin and mucin-domain containing protein 3) is a member of the TIM family of proteins that is preferentially expressed on Th_1_ polarized CD4^+^ and CD8^+^ T cells. Recent studies indicate that TIM-3 serves as a negative regulator of T cell function (i.e. T cell dependent immune responses, proliferation, tolerance, and exhaustion). Despite having no recognizable inhibitory signaling motifs, the intracellular tail of TIM-3 is apparently indispensable for function. Specifically, the conserved residues Y265/Y272 and surrounding amino acids appear to be critical for function. Mechanistically, several studies suggest that TIM-3 can associate with interleukin inducible T cell kinase (ITK), the Src kinases Fyn and Lck, and the p85 phosphatidylinositol 3-kinase (PI3K) adaptor protein to positively or negatively regulate IL-2 production *via* NF-κB/NFAT signaling pathways. To begin to address this discrepancy, we examined the effect of TIM-3 in two model systems. First, we generated several Jurkat T cell lines stably expressing human TIM-3 or murine CD28-ECD/human TIM-3 intracellular tail chimeras and examined the effects that TIM-3 exerts on T cell Receptor (TCR)-mediated activation, cytokine secretion, promoter activity, and protein kinase association. In this model, our results demonstrate that TIM-3 inhibits several TCR-mediated phenotypes: i) NF-kB/NFAT activation, ii) CD69 expression, and iii) suppression of IL-2 secretion. To confirm our Jurkat cell observations we developed a primary human CD8^+^ cell system that expresses endogenous levels of TIM-3. Upon TCR ligation, we observed the loss of NFAT reporter activity and IL-2 secretion, and identified the association of Src kinase Lck, and PLC-γ with TIM-3. Taken together, our results support the conclusion that TIM-3 is a negative regulator of TCR-function by attenuating activation signals mediated by CD3/CD28 co-stimulation.

## Introduction

Immune check-point receptors expressed on T cells have emerged as important targets for the development of cancer immunotherapies (rev. in [1, 2]). In response to viral or bacterial antigens, the concerted interplay between effector CD8^+^, antigen-expressing, cytotoxic T-lymphocytes, and helper CD4^+^ T cells, ensure clearance of infection. Under physiological conditions, immune checkpoints proteins serve to attenuate and/or eliminate sustained immune cell activation, thus regulating normal immune homeostasis. However, during chronic infections and cancer, a sustained state of T cell dysfunction emerges in which the normal effector functions of individual T cell subsets are lost. Referred to as T cell exhaustion, this phenotypic change is characterized by a gradual loss in cytokine secretion, mainly IFN-γ, TNF-α, IL-2, and an increase in inhibitory receptors, CTLA-4, PD-1, LAG-3, and TIM-3, which eventually results in a loss of function (rev. in [3]). In the context of cancer, the deregulated expression of check-point receptors serves as an important mechanism of cancer cell immune resistance. Much attention has focused on targeting the CTLA-4 and PD-1 pathway, including the receptor and its cognate ligands PD-L1/L2, as potential immunotherapy due mostly in part to its broad expression on immune cells, their function within the tumor microenvironment [4, 5] and its well characterized role in the TCR signaling pathway [6–11].

Several studies have demonstrated that TIM-3 is co-expressed with PD-1, both in the context of virally infected CD8^+^ T cells [12–14] and on tumor-infiltrating lymphocytes in melanoma and leukemia [15–17]. TIM-3 was originally identified on mouse Th1 cells [18] and in humans was shown to be expressed on activated CD4^+^ [19], Th17 [20], CD8^+^ T cells, and other immune subsets [21]. To date, Galectin-9 has been identified as a ligand for TIM-3. Galectin-9 binding was shown to increase the apoptotic potential on TIM-3^+^, IFN-γ-secreting, murine Th1, but not Th2 cells [22]. However, it is worth noting that in T cells derived from TIM-3 knock-out mice, galectin-9 mediated cell death of Th1 cells was not completely abolished [22]. In other studies involving human T cell lines (Jurkat and MOLT-4), galectin-9 also demonstrated pleiotropic effects including apoptosis, Ca^2+^ flux, and the loss of mitochondrial membrane potential [23]. Although TIM-3 expression was not confirmed in the study by Lu *et al*, our internal results, and of others, suggest that TIM-3 is not endogenously expressed on quiescent Jurkat or MOLT-4 cells raising the distinct possibility that galectin-9 exerts effects through alternative mechanism(s). Moreover, Leitner *et al* [24] showed that the addition of galectin-9 had no effect on apoptosis or proliferation in activated human T cells, which express TIM-3, consistent with previous findings that galectin-9 induced apoptosis is independent of TIM-3 [25]. Other ligands have been shown to bind TIM-3, mainly phosphotidylserine (PS) and HMGB1. When expressed on phagocytic cells, TIM-3 recognizes apoptotic cells expressing PS, thus supporting a role in phagocytosis [26] and its association with HMGB1 has been shown to interfere with nucleic acid-sensing systems [27], both of which are critical mediators of innate immunity. Based on the association of TIM-3 with T cell exhaustion in multiple settings [12, 15, 28], and its co-expression with PD-1, TIM-3 has emerged as a potential target worth investigating for development of an anti-cancer immunotherapy [29, 30] (reviewed in [31, 32]).

In contrast to our understanding of how PD-1 inhibits T cell receptor (TCR) mediated activation (11), surprisingly very little is known about the role of TIM-3 in this process. The cytoplasmic tail of PD-1 contains two structural motifs, an ITIM (immunoreceptor tyrosine-based inhibition motif) and ITAM (immunoreceptor tyrosine-based activation motif). Upon TCR ligation, the phosphatases SHP-1 and SHP-2 are recruited within a signaling complex involving PD-1 which serves as a negative regulator of T cell activation [8]. However, TIM-3 lacks known structural motifs which could serve a similar function as noted for PD-1. Nonetheless, a conserved tyrosine residue, Y265, located within the cytoplasmic tail of TIM-3, was shown to be phosphorylated by inducible T cell kinase (ITK) upon ligation with galectin-9 when TIM-3 was over-expressed in HEK-293T [33]. Disagreeing studies have emerged as to whether TIM-3 activates or suppresses T cell action. Lee *et al*, [34], using a transient murine TIM-3 expressing Jurkat-T cell model system demonstrated that the tyrosine residues Y256/265 (Y265/272 in humans) were critically important for the coupling of downstream signaling kinases, Fyn and PI-3K p85α subunit. The binding of these kinases to TIM-3 served to augment T cell activation which could be blocked by antagonist antibody. Conversely, Lee *et al*, [35] contested this finding by demonstrating that the presence of TIM-3 in both primary human CD4^+^ and TIM-3 over-expressing Jurkat T cells was sufficient to attenuate NFAT activity with concomitant decreased IL-2 secretion. Consistent with both of these findings is the observation that Y265/272 appears to be required for TIM-3 function.

In order to gain a better understanding of TIM-3 function, we examined NFAT/NF-κB reporter activity in both TIM-3 over-expressing, stable Jurkat T cells and MART-1 peptide differentiated primary human CD8^+^ model systems to address the role that TIM-3 serves in regulating TCR signaling. Our results suggest that the expression of TIM-3 attenuates TCR-induced signaling by specifically inhibiting NFAT reporter activity and down-regulation of IL-2 through a complex signaling network involving Lck and PI-3 kinases, and many other scaffold/adaptor proteins.

## Results

### TIM-3 suppresses TCR-mediated NFAT/NF-κB reporter activity

**Fig 1:**
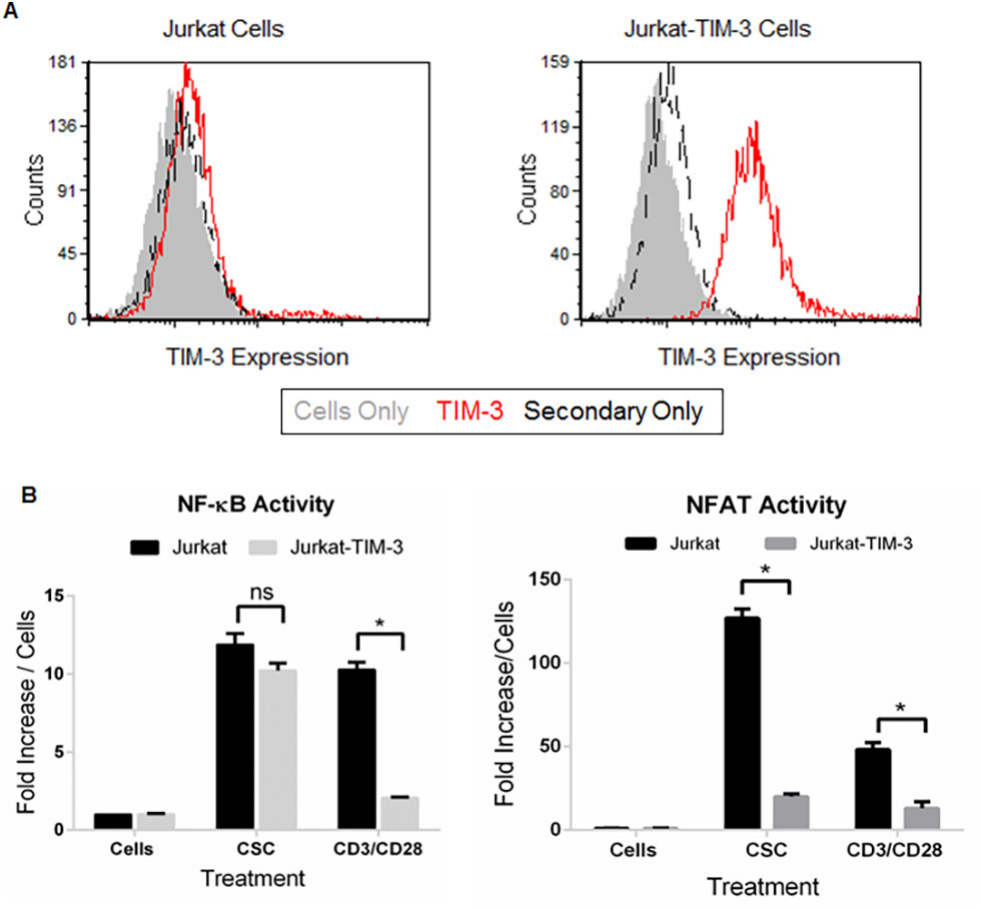
Ectopic TIM-3 expression suppresses TCR-induced activation of NF-κB and NFAT. (A) Flow cytometric analysis of stably transfected, sorted pools shows expression of TIM-3 on the surface of cells. (B) Cells were stimulated with Cell Stimulation Cocktail or anti-CD3/CD28 beads and NF-kB activity, as determined by GFP expression, was measured on an Accumen (left panel). NFAT activation was monitored by transient transfection of cells with NFAT-luciferase reporter plasmid. 72h post-transfection, cells were stimulated and luciferase activity was monitored after 6h using the One-Glow Assay System (right panel). Results are presented as fold change over base line. Results shown in panels B are the average ± SD for quadruplicate samples from a single experiment, representative of at least three independent experiments. (*, P < 0.01; ns: not significant as determined by two-way t-test).

A conflicting paradigm has emerged in which TIM-3 expression has been shown to positively regulate NF-κB [34] and negatively regulate NFAT [35] reporter activity in the Jurkat T cell system. Given this, we sought to clarify these somewhat paradoxical results and address how TIM-3 could intersect signaling pathways originating from the TCR following receptor ligation. Moreover, to avoid aberrant activation associated with transfection methods, we chose to establish a Jurkat-TIM-3 cell line in a stable, lentivirally- transduced, NF-κB-GFP reporter Jurkat T cell line. These cells were transfected with a full-length plasmid encoding TIM-3, sorted for high level of TIM-3 expression, and expanded to obtain a stable Jurkat-NF-κB-GFP-TIM-3 cell line (Fig 1A). Subsequent studies with repeated transfection of siRNA and the removal of antibiotics failed to ablate TIM-3 expression, indicating that this is a stable pool of cells (data not shown). We then addressed the phenotype of TCR-mediated NF-κB activity in the presence of TIM-3. Jurkat-NF-κ B-GFP-TIM-3 were stimulated overnight with cell stimulation cocktail (CSC), a mixture of the phorbol ester, PMA, and the calcium ionophore, ionomycin, or CD3/CD28 beads and GFP expression was monitored by imaging on the Acumen eX3 System and in real-time using the Incucyte (data not shown). As shown in Fig 1B (left panel), stimulation with the non-specific activator PMA/Ionomycin was able to induce GFP expression in both parental and TIM-3 expressing cells. Interestingly, when cells were stimulated with anti-CD3/CD28 beads, we saw a near complete suppression of NF-κB activity as evidenced by the loss of GFP expression in the Jurkat-NF-κB-GFP-TIM-3 cells. Next, we addressed NFAT reporter activity in the same cells as that used to assess NF-κB activity. Because the NFAT-luciferase reporter is a sensitive, enzymatic-based assay, we transiently transfected both parental and the Jurkat-NF-κB-GFP-TIM-3 with the pGL4.3-NFAT-*luc* plasmid and repeated the same stimulation conditions. Optimization of luciferase expression was previously determined and found to be maximal at ~6h post stimulation (data not shown). Consistent with the results for NF-κB, we observed inhibition of NFAT reporter activity when TIM-3 was present (Fig 1B, right panel). Interestingly we saw attenuated NFAT activity in PMA/Ionomycin treated cells and near complete suppression in anti-CD3/anti-CD28 bead stimulated cells. Taken together, our results suggest that TIM-3 interrupts downstream signals originating from the TCR.

### Measurements of Ca^2+^ flux, cytokine changes and T cell activation in response to TCR stimulation

**Fig 2:**
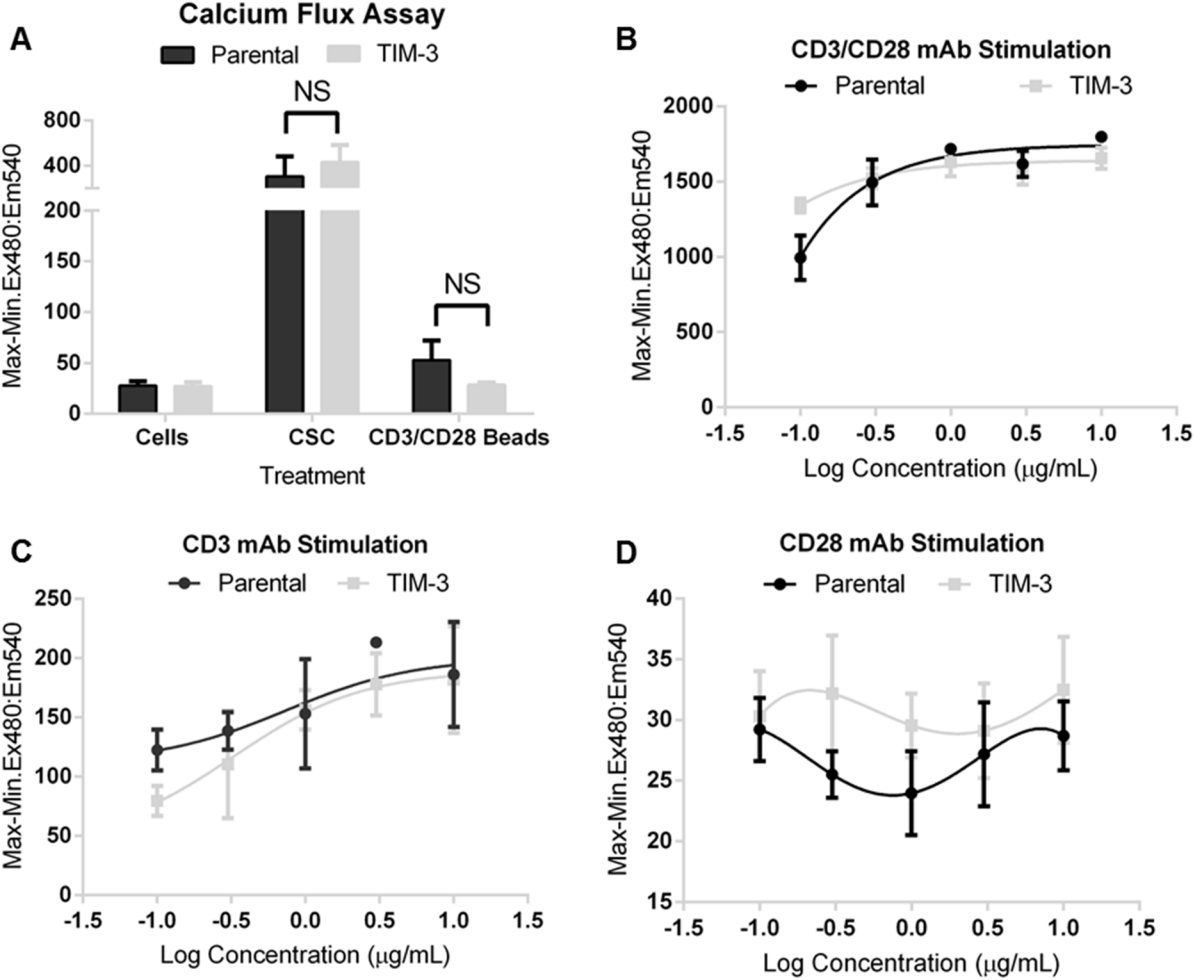
Ectopic Expression of TIM-3 does not suppress CD3/CD28 induced Ca^2+^ Flux. (A) Cells pre-loaded with the fluorescence, calcium-sensitive dye, Cal-520 AM were treated with PMA/Ionomycin, or anti-CD3/CD28 beads, (B) soluble anti-CD3 (OKT3) and anti-CD28 (CD28.2), (C) OKT3 only or (D) CD28.2 only. Calcium flux was measured immediately, in real-time, using the FDSS/μCELL kinetic plate reader. Results are presented as the average ratio of Max-Min for Ex480:Em540, for quadruplicate replicates ± SD, representative of at least three independent experiments.

The release of calcium is required for the activation of both NFAT [36] and NF-κB activation [37]. Moreover, as previously shown, addition of a known TIM-3 ligand, Galectin-9, to polarized murine T_h_1, T cells, induced calcium flux which was substantially reduced in TIM-3 deficient cells [22]. Towards this end, we sought to address whether the attenuation in NFAT/NF-κB reporter activity caused by TIM-3 was due to a lack of Ca^2+^ flux upon TCR ligation. We measured calcium changes using calcium-sensitive dye, Cal-520 AM in both parental and Jurkat-NF-κB-GFP-TIM-3 fluorescent treated with PMA/Ionomycin, or anti-CD3/CD28 beads (Fig 2, panel A), or combined soluble antibodies to CD3 (clone OKT3) and CD28 (clone CD28.2) (Fig 2, panel B). As shown, the presence of TIM-3 did not affect Ca^2+^ flux in cells stimulated with PMA/Ionomycin, anti-CD3/CD28 beads, or anti-CD3 (OKT4). It appears the major contributor to calcium flux change in this assay system was through CD3 (Fig 2, Panel C) and not through CD28 (Fig 2, panel D). Nonetheless, whether we added anti-CD3/CD28 beads or soluble antibodies, we saw similar changes in Ca^2+^ levels in either case, and the presence of TIM-3 did not affect this process as controls were unchanged.

**Fig 3:**
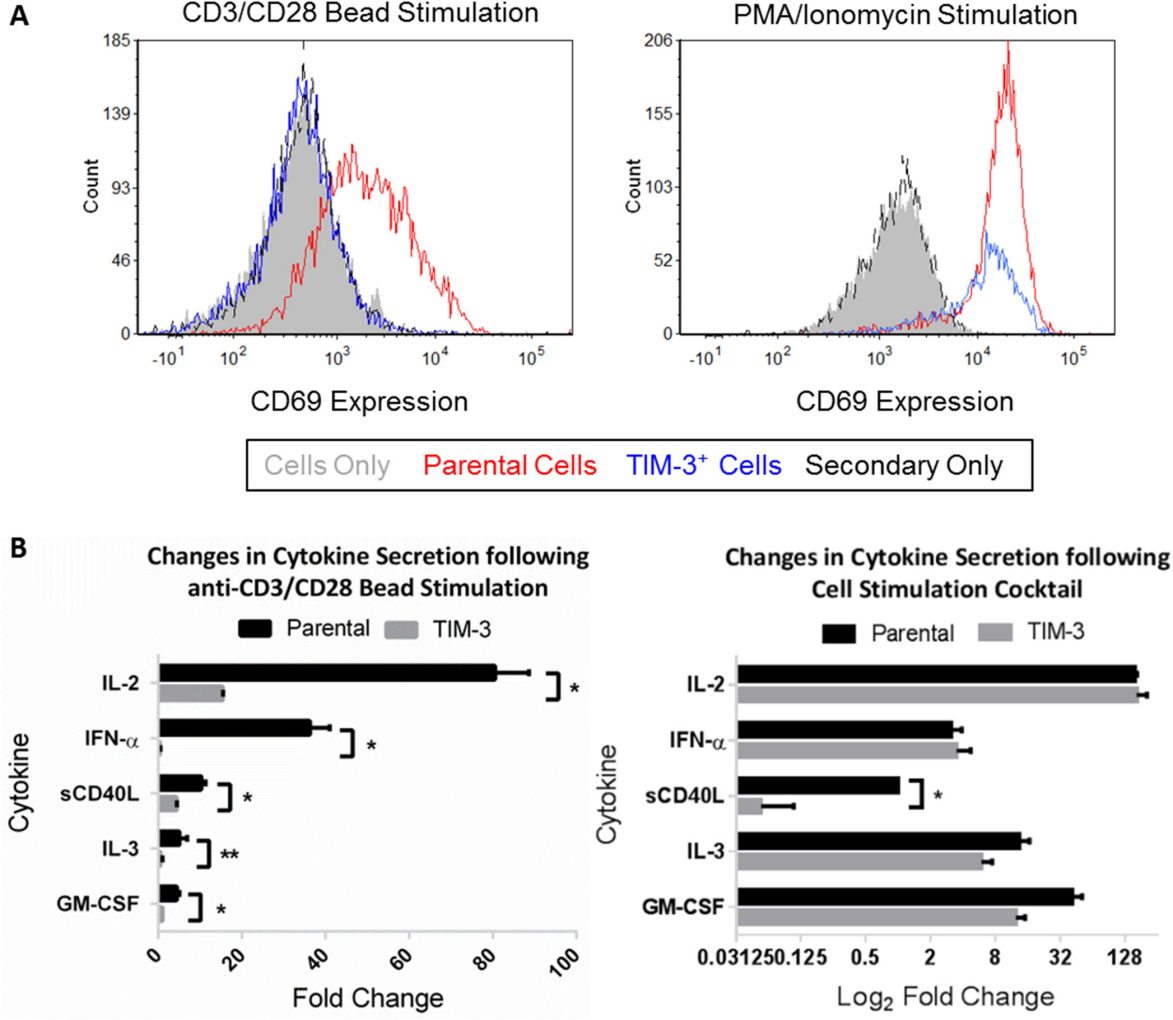
Ectopic Expression of TIM-3 Suppresses CD69 expression and IL-2 secretion. (A) Parental or TIM-3 over-expressing Jurkat T cells were stimulated over night with anti-CD3/CD28 beads or Cell Stimulation Cocktail. Flow cytometric analysis was performed using a CD69-PE conjugated antibody as a positive control and IgG-PE isotype for negative control staining. Red: CD69 for Jurkat-parental; Blue: CD69 for Jurkat-TIM3; Black: Isotype. (B) Cells were stimulated under the same conditions, supernatant was collected and subjected cytokine multiplex analysis. Data represents fold change in observed cytokines for quadruplicate replicates ± SD, representative of at least three independent experiments. (*, P < 0.01; ** P < 0.05 as determined by two-way t-test).

An immediate early marker of T cell activation is the rapid expression of CD69. Given that TIM-3 blocked reporter activity only in the presence of CD3/CD28, we next addressed whether this resulted in a loss of CD69 expression. Rapid expression of the immediate early T cell activation antigen gene, CD69, could help delineate the signaling mechanism as this gene contains responsive elements for several transcription factors including NFAT and NF-κB. In both parental and Jurkat-NF-κB-GFP-TIM-3 cells stimulated with PMA/Ionomycin we observed in the rapid induction of CD69 expression in both parental and TIM-3^+^ (Fig 3A, right panel), however, when cells were stimulated with anti-CD3/CD28 beads (Fig 3A, left panel), only the parental cells were able to induce the expression CD69.

Cytokine expression profile between parental and TIM-3 expressing cells was examined for anti-CD3/CD28 and PMA/Ionomycin stimulated cells. Consistent with previous findings [35] we observed inhibition of IL-2, sCD40L, IFN-α, and IL-3/GM-CSF in TIM-3^+^ cells (Fig 3B, left panel). Likewise, we observed equal expression of IL-2, IFN-α, IL-3, and GM-CSF for both parental and TIM-3 cells with treated with cell stimulation cocktail (Fig 3B, right panel). It is interesting to note that addition of CSC to TIM-3 expressing Jurkat’s reduced the expression of sCD40L. Previous reports have shown that Jurkat cells expressing high levels of sCD40L also showed reduced levels of sCD40L expression following PMA stimulation. The authors suggest the suppression is dependent on protein kinase C activity [38]. For the other cytokines contained in the multiplex panel we did not observe expression of IFN-γ, TNF-α or TNF-β and saw similar levels of expression for IL-1Rα, IL-8, MIP-1α, and MIP-1β following anti-CD3/CD28 bead stimulation (data not shown). Taken together, these results indicate that the TIM-3 suppresses down-stream effector functions following TCR engagement.

### The cytoplasmic tail of TIM-3 is sufficient to block TCR-mediated activation

**Fig 4:**
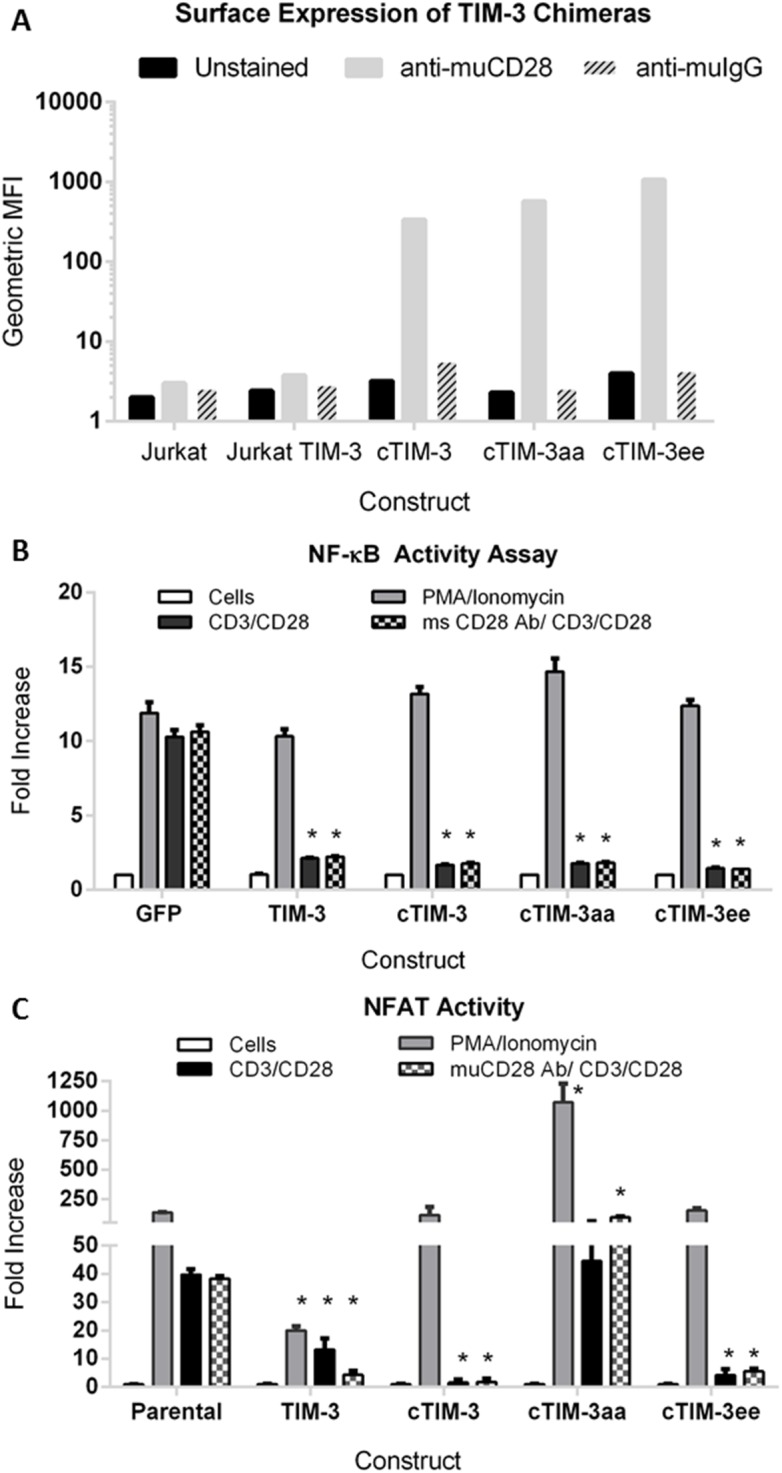
The cytoplasmic domain of human TIM-3 suppresses TCR-induced NF-κB and NFAT Activity. (A) Flow cytometric analysis demonstrating the relative cell surface expression of chimeric protein using an anti-murine CD28 antibody for detection. (B) Cells were stimulated overnight with anti-CD3/CD29 beads or Cell Stimulation Cocktail, NF-κB activity was measured by monitoring GFP expression. (C) Using similar stimulation conditions, NFAT activity was measured 6 hours post-stimulation. Data represents fold change in reporter activity for quadruplicate replicates ± SD, representative of at least three independent experiments. (*, P < 0.01 as determined by two-way t-test).

The use of chimeric receptors to gain an understanding of the importance of specific domains within TIM-3 proteins that are required for function has been previously shown [34, 35]. The combined results of these findings indicate that the cytoplasmic tail and more specifically two conserved tyrosine residues at positions 265 & 272 are required for TIM-3 function. Based on these observations, we generated 3 chimeric TIM-3 molecules (cTIM-3) that expresses the extra-cellular and transmembrane domain of murine CD28 (AA 1–177) fused in-frame with the cytoplasmic tail of human TIM-3 (AA 225–301), 1) wt cTIM-3, 2) a signaling null Y265A/Y272A cTIM-3aa, and 3) a phosphomimetic Y265E/Y272E (cTIM-3ee). To generate stable cell lines, we transfected these plasmids into Jurkat-NF-κB-GFP cells, sorted for high expressors, and expanded the pool to obtain stable Jurkat-NF-κB-GFP chimeric cell lines. Flow cytometry analysis using an anti-murine CD28 antibody confirmed the expression of all of the chimeras on the surface of cells (Fig 4A). It is worth noting that comparable expression levels between full-length TIM-3 (Fig 1A) and the chimeras were achieved. We next addressed whether the presence of the cytoplasmic domain was sufficient to suppress TCR-mediated activation of the NF-κB/NFAT reports. As shown in Fig 4B, anti-CD3/CD28 bead stimulation of the TCR resulted in complete suppression of NF-kB reporter activity for both full-length TIM-3 as well as all of the chimeras. To assess NFAT activity, we transiently expressed the NFAT-luciferase plasmid into these cells and repeated the stimulation. Interestingly, we found that cells expressing the cTIM-3aa were unable to block TCR-induced NFAT activation. All of the receptors expressing either TIM-3, cTIM-3 or cTIM-3ee completely suppressed TCR-mediated NFAT activation (Fig 4C). Interestingly, we found that cells expressing the TIM-3 signaling null chimera (Y265/272A) were unable to block TCR-induced NFAT activation. As a positive control, we observed no suppression of either reporter when cells were treated with PMA/Ionomycin suggesting that the inhibitory properties of TIM-3 are specific for TCR-mediated signaling. It is worth noting that we consistently observed lower NFAT activity in Jurkat-TIM-3 cells treated with either stimulus as compared to NF-kB, suggesting that the TIM-3 plays an important role in regulating NFAT activity.

### Identification of proteins associated with TIM-3 cytoplasmic tail

In an effort to understand and map out the mechanism of TIM-3 mediated signal inhibition, several strategies were employed to identify proteins that interact with the TIM-3 cytoplasmic tail. Previous reports have suggested roles for the p85α phosphatidylinositol 3-kinase (PI-3K) adaptor, the Src kinases Fyn [34] and Lyn [39, 40] and the interleukin inducible T cell kinase (ITK) [33] in mediating TIM-3 function. Similar to the approach taken by Lee, *et al* [34], we generated both biotinylated; tyrosine phosphorylated or unphosphorylated peptides, corresponding to the amino acid sequences surrounding Y265/Y272. These peptides were submitted for Small Molecule-Protein Interaction (SMI), a profiling service that identifies interactions between the peptides and 9,000 human proteins spotted on Life Technologies ProtoArray®. Of the many hits identified by this method, a strong interaction between the phosphorylated TIM-3 peptide and the p85α PI-3K subunit was observed (data not shown). In parallel, we performed an ELISA-based SH2 domain profiling assay that measures the interaction of peptides against an array of 46 SH2 domain-containing proteins spotted on a 96-well plate (Signosis, Santa Clara, CA). Interestingly, this method again identified a positive interaction with the p85α PI-3K subunit as well as other intriguing candidate proteins: the adaptor proteins lymphocyte-specific Adapter Protein (LNK), 3BP2 (SH3BP2) and SH2D2A, the Src kinase Lck, and Phospholipase C (PLCγ1) (data not shown). Even though the SH2 domain of Fyn was contained within this array, we did not observe a positive interaction, this could possibly be due to the fact that we used human TIM-3 peptide sequences vs murine peptide sequences used previously [34].

To confirm that these proteins do in fact interact with TIM-3, we performed co-immuno-precipitation (IP) analyses. TIM-3 peptides were mixed with Jurkat T cell lysate, peptide complexes were pulled down using magnetic streptavidin-beads and subjected to western blot analysis using capillary electrophoresis on the Peggy system (Protein Simple, San Jose, CA). The analyses were expanded to include not only the putative hits identified through Proto- and SH2- array profiling, but kinases that were known to signal through the TCR. As shown in (S1 Fig), we identified a very large complex of proteins that was found to be associated with TIM-3 peptides. We confirmed the association of p85α PI-3K subunit, 3BP2, SH2D2A, Fyn, Lck, Syk, Akt, ZAP-70, and the MAP kinases p38 and JNK. A weak association was noted for PLCγ1 and LNK. No association was observed for the p85β PI-3K subunit, ERK, or IκB.

**Fig 5:**
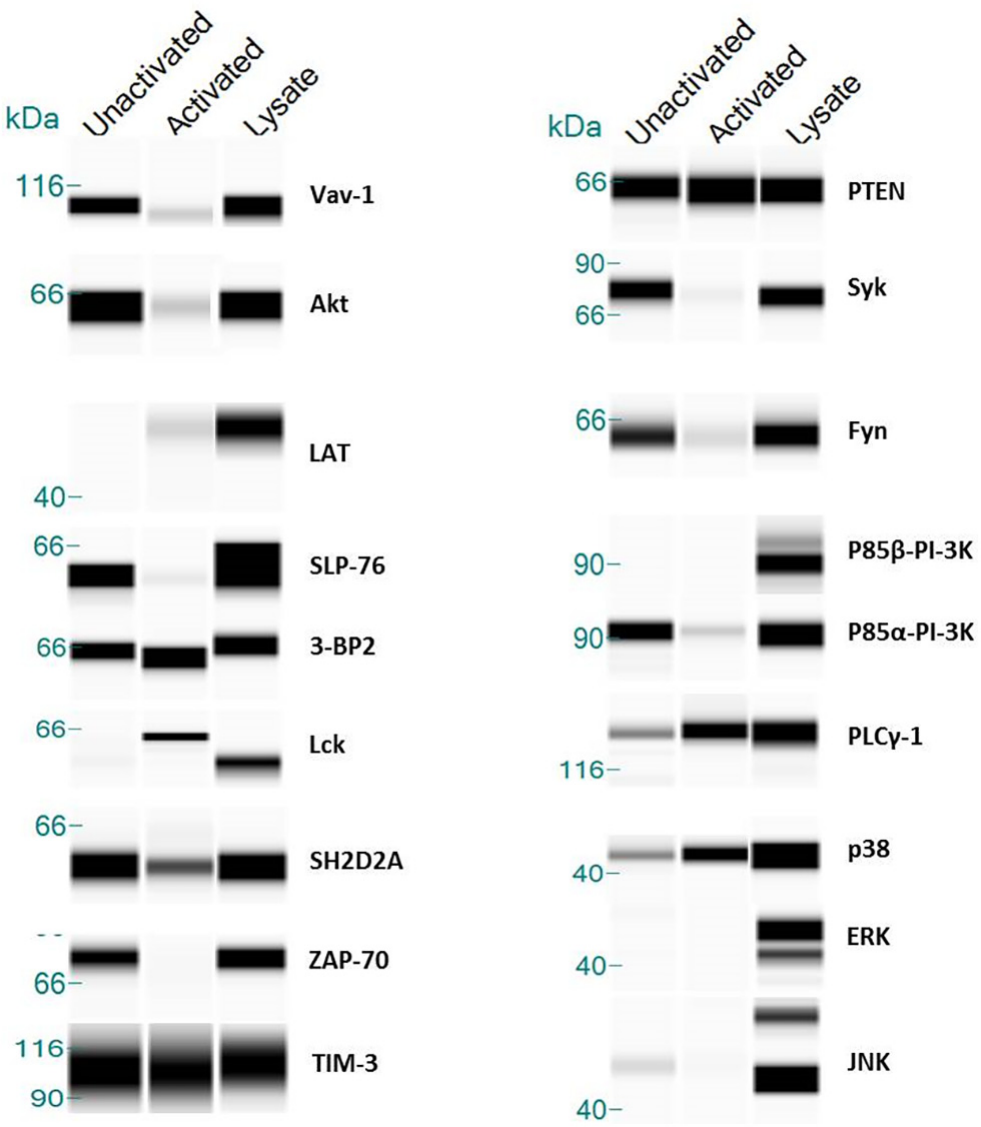
TIM-3 Association with Intracellular Kinases in CD8^+^/MART-1^+^ T cells. TIM-3 co-immunoprecipitation analysis of unactivated and 15 min. stimulation with anti-CD3/CD28 beads (activated). Equivalent amounts of protein (~2mg) were co-immunoprecipitated with pAb anti-TIM-3 antibody and western blot was performed using capillary electrophoresis. Cleared lysate served as a loading control for individual antibody reactivity.

Given that the identification of these kinases was performed using peptides, we sought to confirm if these candidate proteins could also be co-immunoprecipitated from primary human CD8^+^/TIM-3^+^ T cells. Moreover, we also wished to assess what effect activation of the TCR with CD3/CD28 beads would cause on the association of these kinases. To generate these cells, CD8^+^ T cells were antigen expanded with melanoma antigen recognized by T cells (MART-1) peptide loaded onto artificial Antigen Presenting Cells aAPCs at a 1:10 ratio of aAPCs: T cells. Following the conclusion of MART-1 stimulation, these cells were analyzed by flow cytometry which indicated that approximately half of the cells were TIM-3^+^, while all of the cells lacked PD-1 expression (data not shown). MART-1^+^ T cells expressing TIM-3 were stimulated for 15 minutes with anti-CD3/CD28 beads and both unstimulated and stimulated cell lysates were collected. TIM-3 complexes were pulled down using a biotinylated, anti-TIM-3 polyclonal antibody coupled to magnetic streptavidin beads and once again subjected to western blot analysis using capillary electrophoresis. Our results identified a very interesting, yet complicated, signaling complex involving TIM-3 and several kinases previously shown to be involved in TCR signaling [41]. In the basal state, we confirmed our results from peptide co-immunoprecipitation and identified the association of Vav-1, Akt, SLP-76, ZAP-70, Syk, P85α-PI-3K, Fyn, and the adaptor proteins 3-BP2 and SH2D2A (T cell specific adaptor protein) with TIM-3 (Fig 5). Conversely, analysis of lysates from T cells that were activated through ligation of the TCR with anti-CD3/CD28 beads exhibited a much different association pattern. First, the kinases associated with TIM-3 under basal conditions (Vav-1, Akt, SLP-76, ZAP-70, Syk, P85α-PI-3K, Fyn), no longer interacted with TIM-3. Second, activation resulted in the recruitment of the Src family kinase, Lck, as well as enhanced association of PLC-γ1 with TIM-3. As noted previously [34], we also did not see association of TIM-3 with P85β-PI-3K subunit. Taken together, these findings are intriguing. Thus, it appears, the primary step involved in the activation of the TCR is Lck-induced phosphorylation of ITAM motifs within the CD3 subunits of the TCR complex. The extent of ITAM phosphorylation is dependent upon Lck availability, which is tightly regulated by several proteins, including SH2D2A [41]. Following phosphorylation of CD3, recruitment of PLC-γ1 mediates Ca^2+^ and diacylglyercol-induced responses resulting in secondary messenger formation required for NFAT and AP-1 activity [42]. Therefore the sequestration of both Lck and PLC-γ1 by TIM-3 could serve as an inhibitory mechanism in which TIM-3 negatively regulates critically important mediators following TCR activation.

### TIM-3 Mediated Suppression of NF-kB and NFAT activity in differentiated, primary human T cells

**Fig 6:**
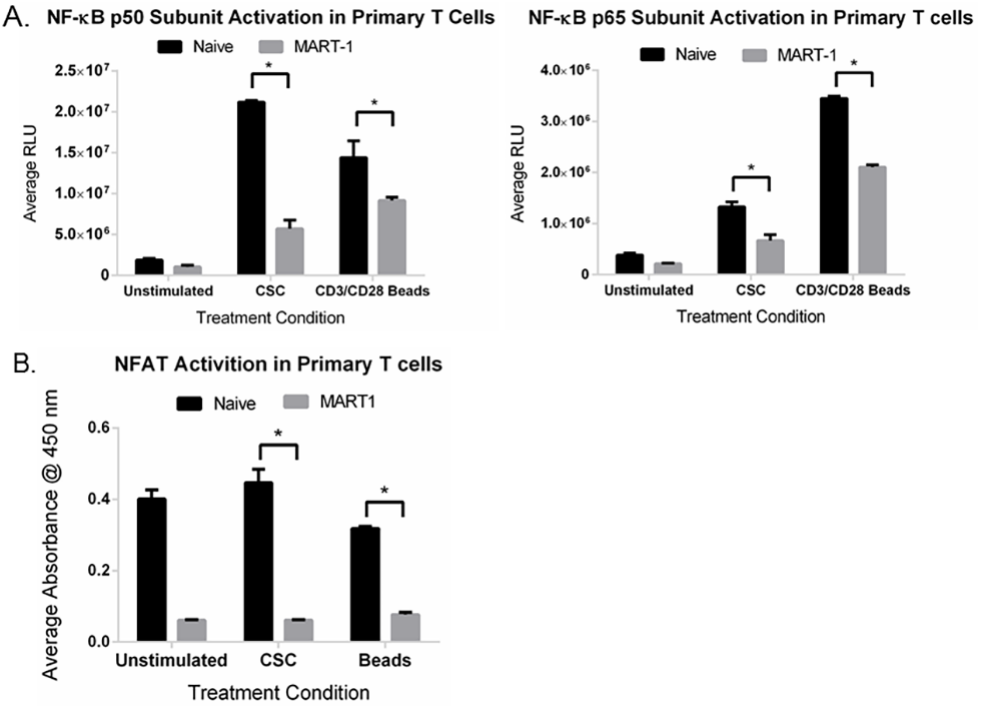
TCR-induced NF-κB/NFAT promoters and cytokine secretion in CD8^+^/MART-1^+^ T cells. Cells were stimulated overnight with cell stimulation cocktail (CSC) or anti-CD3/CD28 beads. Cell supernatents and protein lysates were collected. To evaluate promoter activity, lysates were subjected to (A) NF-kB (left panel: p50 sub-unit; right panel: p65 sub-unit) or (B) NFAT analysis. Data represents fold change in reporter activity for quadruplicate replicates ± SD. (*, P < 0.01 as determined by two-way t-test).

Previous studies indicated that tumor infiltrating lymphocytes (TILs) from mice bearing solid tumors expressed TIM-3 within the CD8^+^ fraction. Moreover, all of these TILs expressed PD-1 and had an impaired ability to proliferate and produce IL-2, TNF-α and IFN-γ [43]. These findings were also confirmed in a mouse model of acute myelogenous leukemia [15]. Given the importance of the NFAT and NF-κB transcription factors in TCR-induced signaling and the combined observations that TIM-3 expressing cells have decreased IL-2 expression and NFAT activity in PMA stimulated primary and Jurkat T cells [35], we evaluated whether TIM-3 suppresses transcriptional reporter activity in TIM-3^+^, primary human CD8^+^ T cells. To test this hypothesis, we assessed the activity of NFAT/NF-κB promoters and downstream IL-2, TNF-α, and IFN-γ secretion in TIM-3^+^ cells as compared to naïve, TIM-3^-^ T cells. To assess reporter activation we utilized chemiluminesence transcription factor assays to measure activity of both the NF-κB p50/p65 subunits and NFAT-1. Briefly, naïve donor CD8^+^ and cognate MART-1 cells were stimulated with PMA/Ionomycin or anti-CD3/CD28 beads, lysates were collected, and reporter activity was assessed. We examined the activity of both the p50 (Fig 6A) and p65 (Fig 6B) subunits of NF-kB and observed a significant decrease in NF-kB activity in the MART-1 cells, consistent with our observations in the Jurkat-TIM-3 reporter system. Next we examined the activity of NFAT in a similar type of reporter assay. The results demonstrated that TIM-3 completely suppressed all NFAT activity to near unstimulated control levels in both PMA/Ionomycin- and anti-CD3/CD28 bead-stimulated MART-1 cells (Fig 6C).

**Fig 7:**
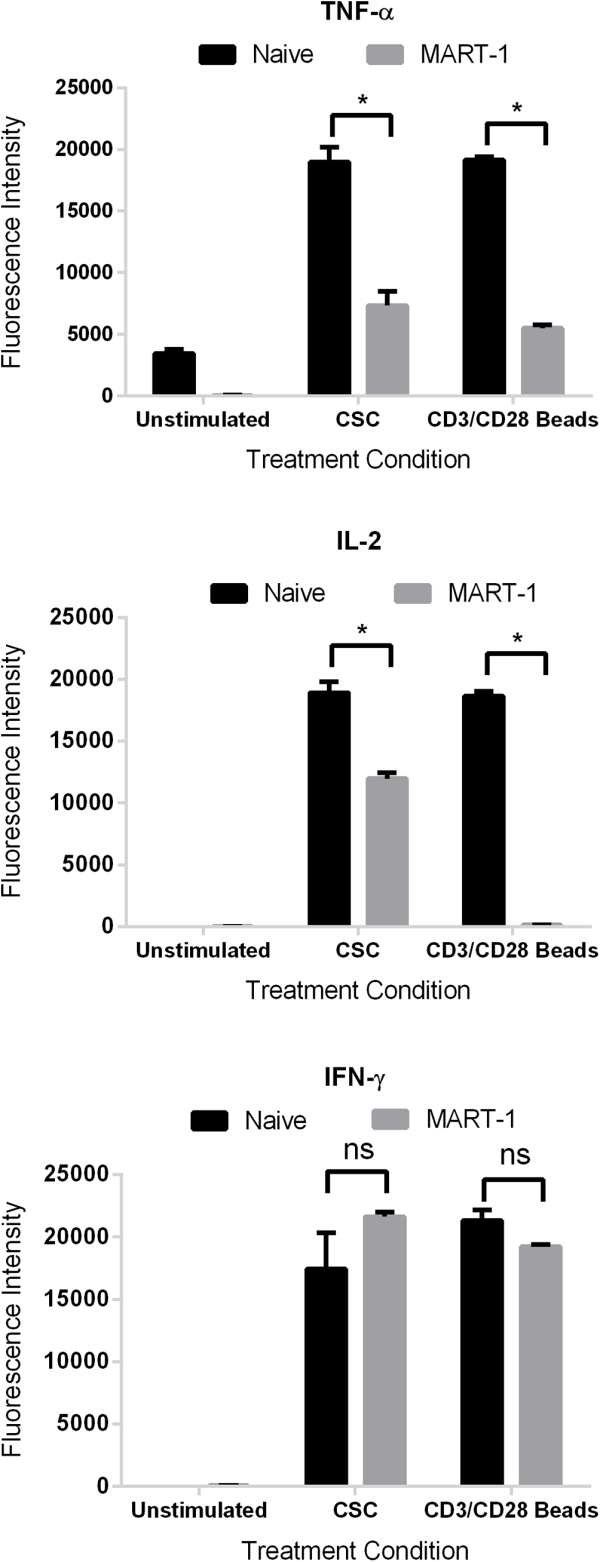
(C) Supernatents from stimulated cells were subject to multiplex analysis for evaluation of cytokine secretion. Data shown for fold change in TNF-α, IFN-γ, and IL-2 secretion, relative to unstimulated control cells, for quadruplicate replicates, ± SD. (*, P < 0.01 as determined by two-way t-test).

NFAT activation is mediated predominantly through cytokine signaling [36]. The promoter for IL-2 harbors two high affinity NFAT-binding sites [44]. Given the lack of NFAT activation in TCR activated, TIM-3^+^ cells, we next addressed if this resulted in the downstream suppression of cytokine secretion. Supernatants from anti-CD3/CD28 bead stimulated cells were collected and subjected to Multiplex Cytokine Analysis (Millipore, Billerica, MA). As shown in Fig 7, naïve T cells exhibited robust induction of IL-2 in contrast to TIM-3^+^ cells where very little IL-2 secretion was observed. Since the T cell culture system requires supplemental IL-2 addition, a potential caveat is that we could be artificially measuring exogenous IL-2. However, this is unlikely to be the case as the basal level of IL-2 in both naïve and MART-1 cells were similar. It is interesting to note that while we saw suppression of IFN-γ in the Jurkat cell system, we observed similar induction, irrespective of stimulus, between naïve and MART-1 cells. Moreover, we observed TNF-α produced by naïve cells as compared to a loss in expression in the MART-1 TIM-3^+^ cells. These results confirm those previously noted on polarized Th1 cells whereby it was demonstrated that human CD4^+^ cells secreted higher levels of IFN-γ and IL-2 when stimulated with anti-CD3/anti-CD28 beads but not TNF-α when TIM-3 was present [19]. Taken together, our results suggest that the expression of TIM-3 serves to attenuate signals emanating from the TCR, specifically TIM-3 blocks NF-κB/NFAT promoter activities resulting in the loss of downstream IL-2 secretion.

## Discussion

The results presented herein demonstrate that TIM-3 serves a negative regulatory role in mitigating activation signals derived from the T cell receptor complex. In two model systems, i) Jurkat T cells over-expressing TIM-3 and ii) primary human T cells endogenously expressing TIM-3, we show that TIM-3 attenuates NFAT reporter activity resulting in the loss of IL-2 secretion. Furthermore, these results suggest that mechanistically, upon stimulation with anti-CD3 and anti-CD28 antibodies, TIM-3 is able to sequester the available pools of Lck and PLC-γ making them unavailable to carry out the necessary activation steps required for full TCR signaling. Given that treatment of cells with anti-CD3 or anti-CD28 resulted in measurable changes in calcium, irrespective of TIM-3 expression, our data are consistent with the model that TIM-3 does not interact directly with CD3 or CD28 but rather regulates TCR function through interactions involving intracellular kinases. This was further supported by our results showing that the TIM-3 intracellular tail is the minimal requirement to exert these inhibitory effects. In Jurkat T cells over-expressing a murine CD28 ECD fused in-frame with the human TIM-3 tail, the overall phenotype is similar to cells over-expressing full-length human TIM-3. More importantly, we demonstrate that the introduction of specific point mutations within the tail at Y265/273A were able to restore NFAT activity, suggesting that these tyrosine residues are critically important determinants for negative regulation by TIM-3. Lastly, our observations that TIM-3 only specifically inhibited anti-CD3/anti-CD28-, and not PMA/Ionomycin-induced activation, lend further support to our conclusion that the expression of TIM-3 on the surface of cells serves a basic function as a negative regulator of TCR signaling.

Evidence supporting the role of TIM-3 as a negative regulator of T cell function was also provided by Sakuishi *et al* [45]. Their results demonstrated that a large majority of murine intra-tumoral FOXP3^+^ regulatory T cells (Tregs) are highly suppressive and co-express TIM3- and PD-1. Those that are mostly TIM-3^+^ were shown to support the development of exhausted CD8^+^ T cells and limit the expansion of effector T cells secreting IFN-γ and TNF-α within the tumor microenvironment. The synergistic effects of Treg depletion plus TIM-3 blockade resulted in significant and sustained tumor regression with concomitant emergence of CD4^+^ and CD8^+^ effector T cells that played a major role in mediating tumor regression. Moreover, in other experimental murine model systems, it was shown that TIM-3 is co-expressed with PD-1 on CD8^+^ tumor-infiltrating lymphocytes (TILs) in mice bearing solid tumors. Dual positive TILs exhibited the most severe exhausted phenotype as defined by a failure to proliferate and an inability to produce IL-2, TNF, and IFN-γ. Furthermore, combined targeting of PD-1 and TIM-3 with mAbs effectively controlled tumor progression than targeting either pathway individually [43]. Furthermore, T cells derived from the cerebrospinal fluid of human patients with multiple sclerosis secreted more IFN-γ than T cells from normal controls. This coincided with lower expression of TIM-3. The use of siRNA to ablate the expression of TIM-3 on CD4^+^ cells, *ex vivo*, resulted in enhanced cell proliferation and IFN-γ secretion [46]. Intracellular staining of CD4^+^ T cells, derived *ex vivo*, stimulated with PMA/Ionomycin, demonstrated that the TIM-3^+^ fraction failed to produce IFN-γ. Moreover, CD4^+^TIM-3^+^ stimulated *in vitro* with anti-CD3/anti-CD28 were found to be unresponsive, as measured by their inability to proliferate or to produce cytokines, mainly IL-2, IFN-γ, or IL-4 [19].

In order to understand how TIM-3 is able to negatively regulate T cell function, several studies have investigated signaling pathways involved in TIM-3-mediated function. The first mechanistic link provided by van de Weyer, *et al*., [33] demonstrated in a HEK 293T dual expression system, the association of TIM-3 with the inducible T cell kinase (ITK) and that ITK was able to specifically phosphorylate TIM-3 at Y265 upon engagement with the TIM-3 ligand, galectin-9. However, the significance of this finding is not yet completely understood, solely because the association between TIM-3 and ITK and the potential functional role of this interaction in T cell model systems has not been demonstrated. Lee *et al*. [35] showed that CD4^+^TIM-3^high^ vs TIM-3^low^ primary human T cells showed reduced IL-2 expression and in stably TIM-3 transfected Jurkat T cells, observed reduced NFAT/AP-1 transcriptional activity which was dependent on regions within the TIM-3 cytoplasmic tail, suggesting that the presence of TIM-3 on the surface of cells negatively regulates activation signals.

A conflicting report supporting the notion that TIM-3 acts as a positive regulator of T cell function was provided by Lee, *et al*. [34]. Using Jurkat T cells expressing murine TIM-3 stably or under the control of a tetracylcine-inducible system, the authors demonstrated that the expression of TIM-3 upregulated NFAT/AP-1 and NF-κB reporter activity upon anti-TCR/CD28 antibody stimulation. Moreover, they suggest that the determinants of reporter activity were dependent on tyrosine residues (Y256/263 in murine TIM-3) and the T cell adaptor proteins ZAP-70 and SLP-76. However, it is worth mentioning that this model system employed the use of almost exclusively murine protein: murine TIM-3 over-expressed in human T cells and the murine T cell clone D10, and retroviral infection of primary murine T cells. Although their results are in conflict with many other published observations suggesting that TIM-3 function as a negative regulator of T cell function, nonetheless, Lee *et al* does provide valuable insight into a potential mechanism through which TIM-3 signals. Their results clearly demonstrate the importance of several critically important kinases required for TCR signaling, mainly the Src kinases Fyn and Lck, the adaptor proteins SLP-76 and ZAP-70, and PLC-γ. In addition, Lee et al [34] also showed that TIM-3 expression increases NF-κB-dependent transcription following TCR and CD28 stimulation. In our primary CD8^+^ T cell model, we noticed that TIM-3 was able to inhibit NF-κB-dependent transcription. Even though we observed complete suppression of NF-κB in an over-expressing Jurkat T-TIM-3 model and not in primary human T cells, one possible explanation for this discrepancy is that Jurkat cells lack PTEN (phosphatase and tensin homologue) and SHIP (SH2-domain-containing inositol polyphosphate 5’ phosphatase) expression (rev. in [47]). Loss of PTEN expression results in activation of the PI-3K pathway and ITK, hence, given the combined observations that these pathways appear to be critically important for TIM-3 signaling one can suffice that studying TIM-3 function in a Jurkat model system has limitations.

Fyn and Lck Src kinases have emerged as important mediators of TIM-3 function. Lee *et al*. [34] demonstrated the importance of these kinases in regulating TIM-3 function. The expression of TIM-3 resulted in the constitutive recruitment of Fyn, and when Lck was co-expressed with TIM-3, stimulation of cells with anti-TCR/anti-CD28 mAbs resulted in more robust and sustained phosphorylation of TIM-3. Moreover, the presence of TIM-3 enhanced the phosphorylation of PLC-γ1 and MAP kinase ERK activation. In our primary human CD8^+^TIM-3+ model system we demonstrate that upon treatment of cells with anti-CD3/anti-CD28 beads, we were able to co-immunoprecipate a complex containing TIM-3, Lck, and PLC-γ1 suggesting that during TCR activation, TIM-3 is recruiting these kinases. This would suggest the ability of TIM-3 to deplete the available intracellular pool of Lck and PLC-γ1 would result in incomplete activation of the TCR by prohibiting the phosphorylation of ITAM motifs on the TCR chains. The inability of TIM-3 to associate with Fyn, in our model system, might provide clues as to how TIM-3 is intersecting TCR signaling. Both Fyn and Lck are critical for TCR signaling, however, each kinase localizes to distinct subcellular compartments. Lck is predominantly associated with the plasma membrane and CD4/CD8, while Fyn is associated with microtubules and the cytoplasmic membrane [48]. Thus it is plausible to assume that membrane proximal signaling events support the association between TIM-3 with Lck.

The first linkage between TIM-3 and the TCR CD3ζ chain in controlling T cell activation was demonstrated by Cho, *et al* [49]. Their findings indicated that tyrosine residues within the cytoplasmic tail of TIM-3 served a critical role in regulating phosphorylation of CD3ζ chain of the TCR, proliferation, and cytotoxicity, thus providing further support for the role of TIM-3 in TCR-mediated signaling events. Further evidence supporting the notion that TIM-3 plays an important role in membrane proximal signaling events within the immunological synapse was shown in primary human TIM-3^+^ T cells [39, 40]. Their results indicated that TIM-3 directly interacts with Lck, however, not the phosho-active form of the kinase required for TCR-induced signaling. Moreover, they suggest that TIM-3 may be recruiting a phosphatase to dampen Lck phosphorylation and that the ratio of inactive vs active Lck within the synapses attenuates TCR-induced signals, thus providing a form of regulatory control over Lck and TCR function [40]. In a retroviral system in which human leukocyte antigen B (HLA-B)-associated transcript 3 (Bat3) was over-expressed in activated primary human Th1 cells, Bat3 was able to bind and recruit active Lck to the TIM-3 tail. Treatment of cells with a TIM-3-specific antibody abrogated the interaction between Bat3 and Lck. Interestingly, when cells were treated with anti-CD3 and–CD28 antibodies, a substantial amount of catalytically inactive Lck accumulated with TIM-3 in Bat3-deficient cells. Interestingly, Bat3 mRNA expression was found to be reduced in TIM-3^+^PD-1^+^ exhausted CD4^+^ T cells from HIV-1 infected individuals. Taken together the authors conclude that TIM-3 forms an intramolecular complex with Bat3 and the active form of Lck to promote T cell signaling with a loss of Bat3 resulting in the accumulation of inactive Lck, defective IL-2 production, and consequentially impaired TCR function [39].

Our results and those of others describe a mechanism suggesting that TIM-3 is recruited to the immunological synapse of T cells. Once there, the intracellular tail of TIM-3 is able to sequester Lck which results in preventing its association with the TCR or blocking its conversion to a catalytically active state. Either outcome ensures that the critically important initial step required for phosphorylation of ITAMs on the TCR sub-unit is not completed. Further studies using TIM-3 tail mutants to identify residues that are required for Lck and PLC-γ1 interaction that functionally rescue this inhibitory phenotype are warranted and currently in progress.

## Materials and Methods

### Cells and antibodies

NF-kB/Jurkat/GFP Transcriptional Reporter Cell Line was obtained from System Biosciences (Mountain View, CA). Leukocytes from normal healthy blood donors were obtained by Biological Specialty Corporation (Colmar, PA) through automated leukapheresis. All healthy volunteers are required to provide informed consent prior to donation. CD8^+^ T cells were isolated through negative selection using a CD8^+^ T Cell isolation kit (Miltenyi Biotech, San Diego, CA). The following antibodies were used for western analysis: total forms of PTEN, P85α- and P85β-PI3K, TIM-3, ERK, AKT, SH2D2A, LCK, Syk, PLC-γ1, JNK, ZAP70, p38, LNK, BMX, IκBα, and donkey-anti-goat IgG HRP-conjugated (R&D Systems, Minneapolis, MN), SH3BP2 (Bethyl Laboratories, Montgomery, TX), LAT, Fyn, SLP76, GRB10, β-Actin (Cell Signaling, Beverly, MA), goat anti-mouse IgG HRP-conjugated, donkey-anti-sheep-F(ab')2-horseradish peroxidase secondary antibody (Jackson ImmunoResearch Laboratories, West Grove, PA), goat-anti-rabbit HRP-conjugated (Protein Simple, San Jose, CA).

### Co-Immunoprecipitation/Western Blotting

Cells were stimulated in micro-centrifuge tubes at 37°C. Cells were pelleted at 12,000 rpm for 30s. Pellets were lysed with ice-cold 1.1% OBG buffer lysis buffer (1.1% n-Octyl-beta-D-glucoside, 20mM Tris-HCl, pH 7.5, 15 mM NaCl) supplemented with Roche Complete Mini Tab Protease Inhibitor Cocktail (Nutley, NJ) and phenylmethanesulfonylfluoride (PMSF) (10μM)(Sigma). Lysates were then incubated on ice for 1h and clarified by centrifugation. Equivalent amounts of lysate (approximately 2mg total protein) were subjected to immunoprecipitation. Briefly, anti-human TIM-3 polyclonal antibody (R&D Systems) was added to the lysate (C_f_ = 1μg/mL) and incubated overnight at 4°C with continuous rocking. Mixture was then added to pre-wash MagnaBind Streptavidin Magnetic Beads (Life Technologies, Grand Island, NY) and incubated for 45min at room temperature. Sample was then washed 5x with lysis buffer. To solubilize protein, immune-complexes were boiled for 7 min with 50μL of Buffer Z (Protein Simple) and subjected to capillary electrophoretic western blotting using the Peggy system (Protein Simple) according to manufacturer’s instructions for size separation. For studies involving peptides, biotinylated peptides (1 μM) were premixed with 2mg of cell lysates and immuno-precipitation procedure was followed as stated above.

### SH2 domain and ProtoArray Profiling

SH2 Domain-based RTK Profiling was obtained from Signosis (Santa Clara, CA) and processed according to the manufacturer’s instructions. Biotinylated TIM-3 peptide, both native and tyrosine phosphorylated used to probe the array was generated by Bachem (Torrance, CA) and used at 0.2–10 μM. Small Molecule Profiling against 9000 human proteins printed on the ProtoArray was performed by Life Technologies. Both peptides were used at 2.5 and 25 μM.

### Plasmid Constructs

NCBI reference sequence, NP_116171.3 was used to generate full-length hepatitis A virus cellular receptor 2 (TIM-3). Protein sequence was human codon optimized and submitted for cloning into the pUNDER expression vector (Invitrogen). To generate chimeric murineCD28 and human TIM-3, NCBI reference sequence, NP_031668.3, for murine CD28 was used. Codon optimized sequences corresponding to amino acids 1–177 of the murine CD28 ECD was fused in in-frame with amino acids 225–301 of human TIM-3 tail. Point mutations corresponding to Y265/272A or Y265/272E, were introduced. Synthesis of constructs, validation of sequences, and maxiprep was performed by genewiz (South Plainfield, NJ).

### Transfection

The NF-κB/Jurkat/GFP™ Transcriptional Reporter cell line (System Biosciences, Mountain View, CA) was cultured in RPMI 1640 media (Life Technologies) supplemented with 10% heat- inactivated FBS (Gibco/Life Technologies, Grand Island, NY). Cells were transfected with a plasmid containing TIM-3 using the Amaxa Cell Line Nucleofector Kit V (Lonza, Cologne, Germany). After 48h, the cells were put under selection with Geneticin (600μg/ml) (Life Technologies). Expression was determined by labeling the cells with anti-human TIM-3 pAb (R&D Systems) or anti-murine CD28 (BioLegend, San Diego, CA) followed by a PE-conjugated Donkey-anti-Goat IgG (Jackson ImmunoResearch) and sorted for TIM-3 positive expression on the BD FACSJazz (BD Biosciences, San Jose, CA) cell sorter using BD FACS Software version 1.1.0.84 (BD Biosciences, San Jose, CA). Live cells and singlets were gated on and then sorted based on anti-TIM-3-PE fluorescence for high signal intensity to generate a Jurkat line enriched for high level of TIM-3 expression. The cells were maintained under selection in media containing 600μg/ml Geneticin. Following the expansion of the culture, cells were once again analyzed to validate receptor expression.

### Reporter Assays

NF-kB/Jurkat/GFP and stably transfected NF-kB/Jurkat/GFP/TIM-3 cells were plated at 2.5e4 cells/well in 96-well Microtest plates (BD Falcon) in RPMI +Glutamax Phenol Red -Free media (Life Technologies). Cells were stimulated overnight at 37°C with either Cell Stimulation Cocktail (CSC) containing phorbol myristate acetate (PMA) and ionomycin (eBioscience, San Diego, CA) or Human T-Activator CD3/CD28 Dynabeads at a ratio of 10:1 (beads:cells) (Life Technologies) and NF-κB reporter activity was assessed by imaging on the Acumen eX3 (TTP Labtech, Cambridge MA). To determine NFAT activity, cells were transiently transfected with 2μg of pGL4.30 (Luc2P/NFAT-RE/Hygro) (Promega, Madison, WI) per 10^6^ cells and allowed to recover for 72h. Cells were stimulated as described and luciferase activity was assessed using the One-Glo luciferase assay system (Promega) and measured on an Envision (Perkin Elmer, Waltham, MA).

### Immunofluorescence Analysis

Staining was performed on an aliquot of cells (2 x 10^5^) from the GFP Reporter Assay. The cells were incubated for 1hr with PerCP/Cy5.5 anti-human CD69 Clone FN50 (BioLegend) at a 1ug/ml concentration. PerCP/Cy5.5 Mouse IgG1kappa Isotype Ctrl Clone MOPC-21 (BioLegend) was used as the control antibody. The cells were analyzed on the BD LSRFortessa (BD Biosciences).

### Cytokine Determination

Supernatants from stimulated Jurkat cells were evaluated for cytokine concentrations using the MILLIPLEX MAP Human Cytokine/Chemokine Magnetic Bead Panel pre-mixed 41-plex Immunology and ssupernatants from primary human CD8+ and MART-1+ T cells were evaluated using the MILLIPLEX MAP Human High Sensitivity T Cell Panel pre-mixed 13-plex Immunology Multiplex assay according to the manufacturers’ instructions (MerckMillipore, MA). Both were analyzed on a BioPlex Multiplex System (Bio-Rad, Hercules, CA)

### NF-κB/NFAT Transcription Factor Analysis

Naïve Human CD8^+^ cells from normal healthy blood donors (Biological Specialty Corp) and MART-1 expanded cells were stimulated overnight at 37°C with either Cell Stimulation Cocktail (CSC) (eBioscience) or Human T-Activator CD3/CD28 Dynabeads (Life Technologies). The cells were harvested and lysed with RIPA buffer (Pierce). The protein concentration was determined by a BCA assay (Life Technologies). 1 μg of the samples were assayed using the Transcription Factor Kits for NF-κB subunits (Life Technologies) and NFAT (Active Motif, Carlsbad, CA).

### Generation of CD8^+^ MART-1 peptide differentiated T cells aAPC Induction

Artificial antigen presenting Drosophila cells (aAPC) were cultured in Express Five Medium (Invitrogen) supplemented with 2mM L-glutamine (Invitrogen) and 200ug/ml Geneticin (Invitrogen) with shaking (100rpm). The cells were cultured every 2–3 days until the viability was >85%. Cells were then washed with PBS, re-suspended in media +5μg/ml UVADEX (Johnson & Johnson) and subjected to cross-linking for 10 minutes at 7.7 Joules/ cm^2^ in a VueLife bag (American Fluoroseal Corporation). An ILT72 UVA Radiometer (Life Technologies) was used for the cross-linking. aAPC cells were loaded with a melanoma antigen recognized by T cells (MART-1) peptide (CS Bio, Menlo Park, CA) by incubating 20 x 10^6^ cells (1 x 10^7^ cells/ml) in Express Five media + 5ug/ml Beta-2M (Janssen in-house) and 0.1μg/ml MART-1 peptide for 4 h at room temperature with mixing every 30 minutes. CD8^+^ T cells were incubated at 37°C for 6 days with Mart-1 peptide loaded aAPCs (1:10 ratio of aAPCs: T cells) and 25ng/ml IL-21 (PeproTech, Rocky Hill, NJ). On day 6, 20 U/ml of IL-2 and 30U/ml IL-7 (PeproTech, Rocky Hill, NJ) were added to the cells and incubated for an additional 2 days. On day 8, re-stimulation was restarted following the procedure stated above and continued until day 14. Flow cytometric analysis was performed to determine viability and TIM-3 receptor expression.

### Statistical analysis

Data were analysed using the unpaired two-tailed Student's *t*-test. *P*-values ≤0.05 or ≤0.01 were considered to be statistically significant.

## Supporting Information

S1 Fig Association of Intracellular Kinases with TIM-3 intracellular tail peptides. Co-immunoprecipitation analysis of Jurkat cell lysate was examined using biotinylated peptides corresponding to the intracellular tail of human TIM-3 (sequence: biotin-SEENIYTIEENVYEVEEP). Where indicated, the tyrosine residues were phosphorylated within the peptide. Protein (~2mg) was co-immunoprecipitated with peptide (1μM) western blot was performed using capillary electrophoresis on the Peggy System. Cleared lysate served as a loading control for individual antibody reactivity.
(TIF)

## References

[1] Pardoll DM. The blockade of immune checkpoints in cancer immunotherapy. Nature reviews Cancer. 2012;12(4):252–64. 10.1038/nrc3239 . 22437870 PMC4856023

[2] Nirschl CJ, Drake CG. Molecular pathways: coexpression of immune checkpoint molecules: signaling pathways and implications for cancer immunotherapy. Clinical cancer research: an official journal of the American Association for Cancer Research. 2013;19(18):4917–24. 10.1158/1078-0432.CCR-12-1972 23868869 PMC4005613

[3] Wherry EJ. T cell exhaustion. Nature immunology. 2011;12(6):492–9. . 21739672 10.1038/ni.2035

[4] Taube JM, Anders RA, Young GD, Xu H, Sharma R, McMiller TL, et al Colocalization of inflammatory response with B7-h1 expression in human melanocytic lesions supports an adaptive resistance mechanism of immune escape. Science translational medicine. 2012;4(127):127ra37 10.1126/scitranslmed.3003689 22461641 PMC3568523

[5] Dong H, Strome SE, Salomao DR, Tamura H, Hirano F, Flies DB, et al Tumor-associated B7-H1 promotes T-cell apoptosis: a potential mechanism of immune evasion. Nature medicine. 2002;8(8):793–800. 10.1038/nm730 . 12091876

[6] Parry RV, Chemnitz JM, Frauwirth KA, Lanfranco AR, Braunstein I, Kobayashi SV, et al CTLA-4 and PD-1 receptors inhibit T-cell activation by distinct mechanisms. Molecular and cellular biology. 2005;25(21):9543–53. 10.1128/MCB.25.21.9543-9553.2005 16227604 PMC1265804

[7] Freeman GJ, Long AJ, Iwai Y, Bourque K, Chernova T, Nishimura H, et al Engagement of the PD-1 immunoinhibitory receptor by a novel B7 family member leads to negative regulation of lymphocyte activation. The Journal of experimental medicine. 2000;192(7):1027–34. 11015443 10.1084/jem.192.7.1027 PMC2193311

[8] Chemnitz JM, Parry RV, Nichols KE, June CH, Riley JL. SHP-1 and SHP-2 associate with immunoreceptor tyrosine-based switch motif of programmed death 1 upon primary human T cell stimulation, but only receptor ligation prevents T cell activation. Journal of immunology. 2004;173(2):945–54. . 15240681 10.4049/jimmunol.173.2.945

[9] Sheppard KA, Fitz LJ, Lee JM, Benander C, George JA, Wooters J, et al PD-1 inhibits T-cell receptor induced phosphorylation of the ZAP70/CD3zeta signalosome and downstream signaling to PKCtheta. FEBS letters. 2004;574(1–3):37–41. 10.1016/j.febslet.2004.07.083 . 15358536

[10] Li J, Jie HB, Lei Y, Gildener-Leapman N, Trivedi S, Green T, et al PD-1/SHP-2 Inhibits Tc1/Th1 Phenotypic Responses and the Activation of T Cells in the Tumor Microenvironment. Cancer research. 2014 10.1158/0008-5472.CAN-14-1215 . 25480946 PMC4315704

[11] Riley JL. PD-1 signaling in primary T cells. Immunological reviews. 2009;229(1):114–25. 10.1111/j.1600-065X.2009.00767.x 19426218 PMC3424066

[12] Jones RB, Ndhlovu LC, Barbour JD, Sheth PM, Jha AR, Long BR, et al Tim-3 expression defines a novel population of dysfunctional T cells with highly elevated frequencies in progressive HIV-1 infection. The Journal of experimental medicine. 2008;205(12):2763–79. 10.1084/jem.20081398 19001139 PMC2585847

[13] Jin HT, Anderson AC, Tan WG, West EE, Ha SJ, Araki K, et al Cooperation of Tim-3 and PD-1 in CD8 T-cell exhaustion during chronic viral infection. Proceedings of the National Academy of Sciences of the United States of America. 2010;107(33):14733–8. 10.1073/pnas.1009731107 20679213 PMC2930455

[14] Golden-Mason L, Palmer BE, Kassam N, Townshend-Bulson L, Livingston S, McMahon BJ, et al Negative immune regulator Tim-3 is overexpressed on T cells in hepatitis C virus infection and its blockade rescues dysfunctional CD4+ and CD8+ T cells. Journal of virology. 2009;83(18):9122–30. 10.1128/JVI.00639-09 19587053 PMC2738247

[15] Zhou Q, Munger ME, Veenstra RG, Weigel BJ, Hirashima M, Munn DH, et al Coexpression of Tim-3 and PD-1 identifies a CD8+ T-cell exhaustion phenotype in mice with disseminated acute myelogenous leukemia. Blood. 2011;117(17):4501–10. 10.1182/blood-2010-10-310425 21385853 PMC3099570

[16] Fourcade J, Sun Z, Pagliano O, Chauvin JM, Sander C, Janjic B, et al PD-1 and Tim-3 regulate the expansion of tumor antigen-specific CD8(+) T cells induced by melanoma vaccines. Cancer research. 2014;74(4):1045–55. 10.1158/0008-5472.CAN-13-2908 24343228 PMC3952491

[17] Fourcade J, Sun Z, Benallaoua M, Guillaume P, Luescher IF, Sander C, et al Upregulation of Tim-3 and PD-1 expression is associated with tumor antigen-specific CD8+ T cell dysfunction in melanoma patients. The Journal of experimental medicine. 2010;207(10):2175–86. 10.1084/jem.20100637 20819923 PMC2947081

[18] Monney L, Sabatos CA, Gaglia JL, Ryu A, Waldner H, Chernova T, et al Th1-specific cell surface protein Tim-3 regulates macrophage activation and severity of an autoimmune disease. Nature. 2002;415(6871):536–41. 10.1038/415536a . 11823861

[19] Hastings WD, Anderson DE, Kassam N, Koguchi K, Greenfield EA, Kent SC, et al TIM-3 is expressed on activated human CD4+ T cells and regulates Th1 and Th17 cytokines. European journal of immunology. 2009;39(9):2492–501. 10.1002/eji.200939274 19676072 PMC2759376

[20] Nakae S, Iwakura Y, Suto H, Galli SJ. Phenotypic differences between Th1 and Th17 cells and negative regulation of Th1 cell differentiation by IL-17. Journal of leukocyte biology. 2007;81(5):1258–68. 10.1189/jlb.1006610 . 17307864

[21] Anderson AC, Anderson DE, Bregoli L, Hastings WD, Kassam N, Lei C, et al Promotion of tissue inflammation by the immune receptor Tim-3 expressed on innate immune cells. Science. 2007;318(5853):1141–3. 10.1126/science.1148536 . 18006747

[22] Zhu C, Anderson AC, Schubart A, Xiong H, Imitola J, Khoury SJ, et al The Tim-3 ligand galectin-9 negatively regulates T helper type 1 immunity. Nature immunology. 2005;6(12):1245–52. 10.1038/ni1271 . 16286920

[23] Lu LH, Nakagawa R, Kashio Y, Ito A, Shoji H, Nishi N, et al Characterization of galectin-9-induced death of Jurkat T cells. Journal of biochemistry. 2007;141(2):157–72. 10.1093/jb/mvm019 . 17167046

[24] Leitner J, Rieger A, Pickl WF, Zlabinger G, Grabmeier-Pfistershammer K, Steinberger P. TIM-3 does not act as a receptor for galectin-9. PLoS pathogens. 2013;9(3):e1003253 10.1371/journal.ppat.1003253 23555261 PMC3605152

[25] Su EW, Bi S, Kane LP. Galectin-9 regulates T helper cell function independently of Tim-3. Glycobiology. 2011;21(10):1258–65. 10.1093/glycob/cwq214 21187321 PMC3167474

[26] Nakayama M, Akiba H, Takeda K, Kojima Y, Hashiguchi M, Azuma M, et al Tim-3 mediates phagocytosis of apoptotic cells and cross-presentation. Blood. 2009;113(16):3821–30. 10.1182/blood-2008-10-185884 . 19224762

[27] Chiba S, Baghdadi M, Akiba H, Yoshiyama H, Kinoshita I, Dosaka-Akita H, et al Tumor-infiltrating DCs suppress nucleic acid-mediated innate immune responses through interactions between the receptor TIM-3 and the alarmin HMGB1. Nature immunology. 2012;13(9):832–42. 10.1038/ni.2376 22842346 PMC3622453

[28] Blackburn SD, Shin H, Haining WN, Zou T, Workman CJ, Polley A, et al Coregulation of CD8+ T cell exhaustion by multiple inhibitory receptors during chronic viral infection. Nature immunology. 2009;10(1):29–37. 10.1038/ni.1679 19043418 PMC2605166

[29] Ngiow SF, von Scheidt B, Akiba H, Yagita H, Teng MW, Smyth MJ. Anti-TIM3 antibody promotes T cell IFN-gamma-mediated antitumor immunity and suppresses established tumors. Cancer research. 2011;71(10):3540–51. 10.1158/0008-5472.CAN-11-0096 . 21430066

[30] Kikushige Y, Shima T, Takayanagi S, Urata S, Miyamoto T, Iwasaki H, et al TIM-3 is a promising target to selectively kill acute myeloid leukemia stem cells. Cell stem cell. 2010;7(6):708–17. 10.1016/j.stem.2010.11.014 . 21112565

[31] Ngiow SF, Teng MW, Smyth MJ. Prospects for TIM3-Targeted Antitumor Immunotherapy. Cancer research. 2011;71(21):6567–71. 10.1158/0008-5472.CAN-11-1487 . 22009533

[32] Anderson DE. TIM-3 as a therapeutic target in human inflammatory diseases. Expert opinion on therapeutic targets. 2007;11(8):1005–9. 10.1517/14728222.11.8.1005 . 17665973

[33] van de Weyer PS, Muehlfeit M, Klose C, Bonventre JV, Walz G, Kuehn EW. A highly conserved tyrosine of Tim-3 is phosphorylated upon stimulation by its ligand galectin-9. Biochemical and biophysical research communications. 2006;351(2):571–6. 10.1016/j.bbrc.2006.10.079 . 17069754

[34] Lee J, Su EW, Zhu C, Hainline S, Phuah J, Moroco JA, et al Phosphotyrosine-dependent coupling of Tim-3 to T-cell receptor signaling pathways. Molecular and cellular biology. 2011;31(19):3963–74. 10.1128/MCB.05297-11 21807895 PMC3187355

[35] Lee MJ, Woo MY, Chwae YJ, Kwon MH, Kim K, Park S. Down-regulation of interleukin-2 production by CD4(+) T cells expressing TIM-3 through suppression of NFAT dephosphorylation and AP-1 transcription. Immunobiology. 2012;217(10):986–95. 10.1016/j.imbio.2012.01.012 . 22445722

[36] Macian F. NFAT proteins: key regulators of T-cell development and function. Nature reviews Immunology. 2005;5(6):472–84. 10.1038/nri1632 . 15928679

[37] Dolmetsch RE, Lewis RS, Goodnow CC, Healy JI. Differential activation of transcription factors induced by Ca2+ response amplitude and duration. Nature. 1997;386(6627):855–8. 10.1038/386855a0 . 9126747

[38] Lisowska K, Bryl E, Soroczynska M, Witkowski JM. Modulation of CD40L antigen expression in Jurkat cells: involvement of protein kinase C activity. Folia histochemica et cytobiologica / Polish Academy of Sciences, Polish Histochemical and Cytochemical Society. 2003;41(4):233–5. . 14677764

[39] Rangachari M, Zhu C, Sakuishi K, Xiao S, Karman J, Chen A, et al Bat3 promotes T cell responses and autoimmunity by repressing Tim-3-mediated cell death and exhaustion. Nature medicine. 2012;18(9):1394–400. 10.1038/nm.2871 22863785 PMC3491118

[40] Clayton KL, Haaland MS, Douglas-Vail MB, Mujib S, Chew GM, Ndhlovu LC, et al T cell Ig and mucin domain-containing protein 3 is recruited to the immune synapse, disrupts stable synapse formation, and associates with receptor phosphatases. Journal of immunology. 2014;192(2):782–91. 10.4049/jimmunol.1302663 24337741 PMC4214929

[41] Acuto O, Di Bartolo V, Michel F. Tailoring T-cell receptor signals by proximal negative feedback mechanisms. Nature reviews Immunology. 2008;8(9):699–712. 10.1038/nri2397 . 18728635

[42] Jordan MS, Singer AL, Koretzky GA. Adaptors as central mediators of signal transduction in immune cells. Nature immunology. 2003;4(2):110–6. 10.1038/ni0203-110 . 12555096

[43] Sakuishi K, Apetoh L, Sullivan JM, Blazar BR, Kuchroo VK, Anderson AC. Targeting Tim-3 and PD-1 pathways to reverse T cell exhaustion and restore anti-tumor immunity. The Journal of experimental medicine. 2010;207(10):2187–94. 10.1084/jem.20100643 20819927 PMC2947065

[44] Serfling E, Avots A, Neumann M. The architecture of the interleukin-2 promoter: a reflection of T lymphocyte activation. Biochimica et biophysica acta. 1995;1263(3):181–200. . 7548205 10.1016/0167-4781(95)00112-t

[45] Sakuishi K, Ngiow SF, Sullivan JM, Teng MW, Kuchroo VK, Smyth MJ, et al TIM3FOXP3 regulatory T cells are tissue-specific promoters of T-cell dysfunction in cancer. Oncoimmunology. 2013;2(4):e23849 10.4161/onci.23849 23734331 PMC3654601

[46] Koguchi K, Anderson DE, Yang L, O'Connor KC, Kuchroo VK, Hafler DA. Dysregulated T cell expression of TIM3 in multiple sclerosis. The Journal of experimental medicine. 2006;203(6):1413–8. 10.1084/jem.20060210 16754722 PMC2118310

[47] Abraham RT, Weiss A. Jurkat T cells and development of the T-cell receptor signalling paradigm. Nature reviews Immunology. 2004;4(4):301–8. 10.1038/nri1330 . 15057788

[48] Ley SC, Marsh M, Bebbington CR, Proudfoot K, Jordan P. Distinct intracellular localization of Lck and Fyn protein tyrosine kinases in human T lymphocytes. The Journal of cell biology. 1994;125(3):639–49. 7513706 10.1083/jcb.125.3.639 PMC2119993

[49] Cho JL, Roche MI, Sandall B, Brass AL, Seed B, Xavier RJ, et al Enhanced Tim3 activity improves survival after influenza infection. Journal of immunology. 2012;189(6):2879–89. 10.4049/jimmunol.1102483 22875804 PMC3436990

