# Supplementary figures and images for: TIM-3 Suppresses Anti-CD3/CD28-Induced TCR Activation and IL-2 Expression through the NFAT Signaling Pathway
Source: PLoS One. 2015 Oct 22;10(10):e0140694. doi: 10.1371/journal.pone.0140694 (PMC4619610; doi:10.1371/journal.pone.0140694)

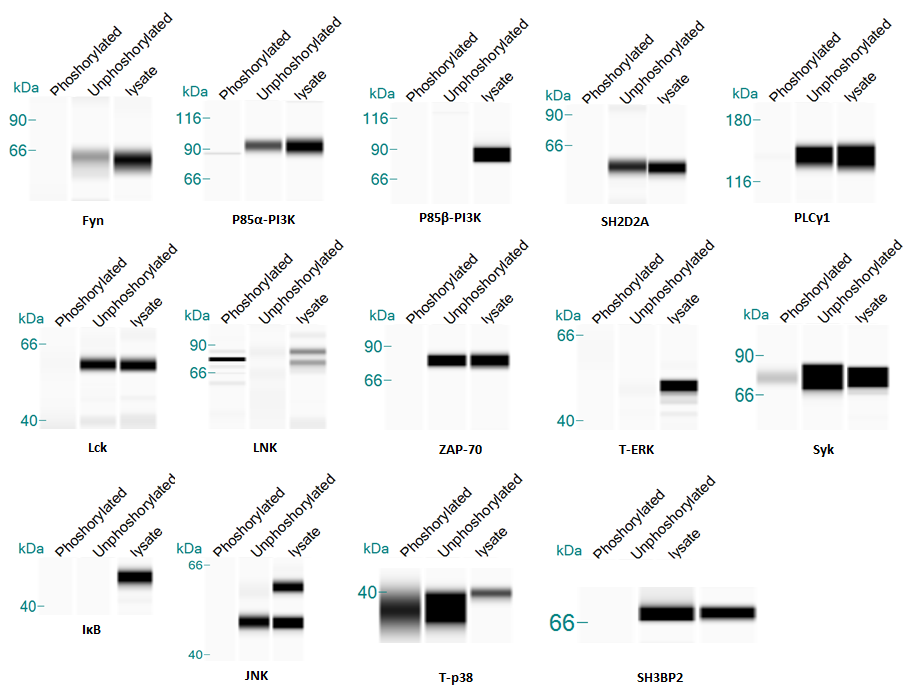

Supplement: S1 Fig — Co-immunoprecipitation analysis of Jurkat cell lysate was examined using biotinylated peptides corresponding to the intracellular tail of human TIM-3 (sequence: biotin-SEENIYTIEENVYEVEEP). Where indicated, the tyrosine residues were phosphorylated within the peptide. Protein (~2mg) was co-immunoprecipitated with peptide (1μM) western blot was performed using capillary electrophoresis on the Peggy System. Cleared lysate served as a loading control for individual antibody reactivity. (TIF) [file pone.0140694.s001.tif]
